# Third-party intervention and post-conflict behaviour in agonistic encounters of pigs (*Sus scrofa*)

**DOI:** 10.1186/s12983-023-00508-w

**Published:** 2023-08-17

**Authors:** Nicole Maffezzini, Simon P. Turner, J. Elizabeth Bolhuis, Gareth Arnott, Irene Camerlink

**Affiliations:** 1 Institute of Genetics and Animal Biotechnology, Polish Academy of Sciences, Jastrzębiec, Poland; 2 Animal Behaviour and Welfare, Animal and Veterinary Sciences Department, Scotland’s Rural College (SRUC), West Mains Rd., Edinburgh, EH9 3JG UK; 3 Adaptation Physiology Group, Department of Animal Sciences, Wageningen University and Research, Wageningen, The Netherlands; 4 Institute for Global Food Security, School of Biological Sciences, Queen’s University, Belfast, BT9 7BL UK

**Keywords:** Animal contest, Aggression, Coalition formation, Conflict resolution, Social behaviour, Social support theory, Third party interaction

## Abstract

**Background:**

Third-party interference in agonistic contests entails a deliberate intervention in an ongoing fight by a bystanding individual (third party) and may be followed by post-conflict social behaviour to provide support to a specific individual. The mechanisms behind third-party intervention are, however, still largely understudied. The aim of this study was to investigate third-party interference, with the predictions that (1) the interferer derives benefits from its action by winning a fight, (2) that patterns of intervention depend on familiarity, (3) that dyadic fights last longer than triadic fights, and (4) that interferers engage in non-agonistic social behaviours afterwards. Pre-pubertal pigs (*Sus scrofa*) (n = 384) were grouped with one familiar and four unfamiliar conspecifics (all non-kin) to elicit contests for dominance rank. Third-party interference was analysed for the first 30 min after grouping, along with the behaviour (nosing or aggression), contest duration, contest outcome, and interferer behaviour after the fight (post-conflict social behaviour).

**Results:**

Three types of interference were observed: non-agonistic involvement (nose contact) by the interferer in a dyadic fight; a triadic fight with each of three contestants fighting one opponent at a time; and triadic fights with two opponents jointly attacking the third one (two-against-one fights). The likelihood of a third-party intervention to occur did not depend on the presence of a familiar animal in the fight. However, once intervention was triggered, interferers attacked unfamiliar fight initiators more than familiar ones. Two-against-one fights lasted longer than other triadic fights and occurred more often when both initial contestants were females. Results of 110 triadic fights (out of 585 fights in total) revealed that interferers were more likely to win compared to the initial opponents at equal body weight. The most common post-conflict behaviour displayed by the interferer was agonistic behaviour towards another group member, independently of familiarity.

**Conclusions:**

The general lack of discrimination for familiarity suggests interference is not driven by support to familiar individuals in pigs. The results show that intervening in an ongoing fight gives the interferer a high chance of contest success and may be a strategy that is beneficial to the interferer to increase its dominance status.

**Supplementary Information:**

The online version contains supplementary material available at 10.1186/s12983-023-00508-w.

## Background

In an agonistic context, third-party interactions occur when a bystander takes part in an on-going fight between two opponents [93]. Third-party interference in ongoing interactions has been described in a few species, mostly primates, social carnivores, and corvids [3, 18, 60, 81]. Interference can be either *impartial*, when the interferer participates in the fight without taking a side, or *partial*, when the interferer preferentially attacks one of the opponents, thus supporting the other (deliberately or not) [5, 30, 92]. The latter scenario includes the case of coalition formation, when at least two individuals coordinate their actions against a third party in an aggressive context [6, 64]). Studies on animal contests have mostly focused on dyadic interactions, and therefore the mechanisms driving the interferer’s participation in a fight are still not clear. Expanding the current knowledge of agonistic scenarios beyond dyadic conflicts is important in order to better understand animal decision making and the evolution of interference behaviour [8].

Contests are inherently costly and pose a risk of serious injuries [73, 76]. Therefore, for interfering behaviour to have evolved, we should expect the intervening individual to gain benefits from participation in contests. By winning, an interferer may directly increase its own dominance status, which in turn may lead to increased resource access or reproductive success [1, 35, 43]. According to the direct benefits theory, we expect (*prediction 1*) that interferers will be more likely to win an encounter than either of the initial opponents (partial intervention with direct benefits).

An alternative to a direct benefit for the interferer, in the form of an increase in dominance status, is that intervention takes place in order to provide aid to kin or a familiar animal, directly benefitting the recipient [49, 74, 82, 86]. In some cases, aiding during an agonistic interaction may be limited to providing social support, meaning the simple presence of a familiar individual or the performance of affiliative social behaviour, both of which are thought to act as a “buffer” against challenging situations, reducing the experience of stress for the recipient animal [98]. On the other hand, social aid in agonistic contexts may also involve the active engagement of a third individual in the fight [6, 69]. If an interferer aims to support a preferred individual which is involved in a fight, the interferer may direct its efforts to stop the lesser preferred opponent. Following inclusive fitness theory [37], animals are more likely to prioritize supporting kin over unrelated individuals [7]. In some group-living species, in-group cooperation occurs between kin as well as non-kin, depending on the strength of the social relationship [79, 89]. In groups of non-kin, we therefore expect that interferers will direct their attacks to the unfamiliar (out-group) opponent in the fighting dyad, thereby potentially supporting a familiar (in-group) individual. We thus expect (*prediction 2*) familiarity to play a role in determining patterns of intervention (partial intervention with indirect benefits). These two outcomes of interference, of gaining a higher dominance status or providing social aid, are not mutually exclusive, but may coexist in the same population and even be achieved by the same individual. Depending on the situation, help may be provided either to social partners or to individuals with a higher social ranking, whose support not only increases the chances of winning and reduces the risk of injury but may also yield benefits later on, for example by reinforcing relationships with more dominant group members [32, 59].

At a group level, third-party intervention may also be an example of prosocial “policing”, having evolved as an effective mechanism to stem the negative effects of a fight by helping terminate the aggression. It may therefore promote cohesion among group members, which in many species is necessary for the individual’s survival and fitness (“group stability hypothesis”) [31, 59]. Policing is an expression of impartial interventions, as the interferer does not take sides with either one of the opponents but targets them both in order to stop the conflict [59, 92]. According to the group stability hypothesis, third-party intervention would decrease the duration of a fight. Therefore, we expect (*prediction 3*) that dyadic fights last longer than triadic fights (with or without the temporary coalition of two individuals) (impartial intervention with indirect benefits).

Triadic interactions may not be limited to the fight but may persist even after. In the specific case of post-conflict affiliation, a bystander approaches one of the opponents while providing positive social contact, and this may constitute an effective way to mitigate the consequences of a conflict in social species, and promote group cohesion [48]. Post-conflict affiliation (i.e., consolation) may be directed towards a winner or loser [12, 19, 83]. Among the possible explanations for post-conflict affiliation is the protection of the loser or other group members from further aggression [21, 66, 97]. Therefore, we further expect (*prediction 4*) that interferers would engage in non-agonistic social behaviours after the conflict.

These predictions were tested in an observational study conducted on 384 pre-pubertal domestic pigs (*Sus scrofa* *domesticus*), in a scenario where pairs of group mates were introduced to two other pairs that they were unfamiliar to. Ungulates are an interesting but understudied taxonomic group for the study of third-party interference [42, 80]. Pigs are a particularly interesting model as they show complex social relationships and, upon encountering unfamiliar conspecifics, engage in intense agonistic interactions, including costly dyadic as well as triadic fights, in order to establish dominance ranks [11, 22, 29]. They also demonstrate advanced socio-cognitive skills (e.g. discriminating conspecifics, deceiving others [57]), engage in contests with clear winners and quantifiable costs [11] and engage, post-conflict, in non-random solicited and unsolicited interactions with third parties [22]. The aim of the current study is, therefore, to investigate third-party interference and its drivers in an agonistic context in domestic pigs, and to assess the potential benefits that animals may derive from interfering.

## Results

### Occurrence and general characteristics of third-party interactions

**Fig. 1:**
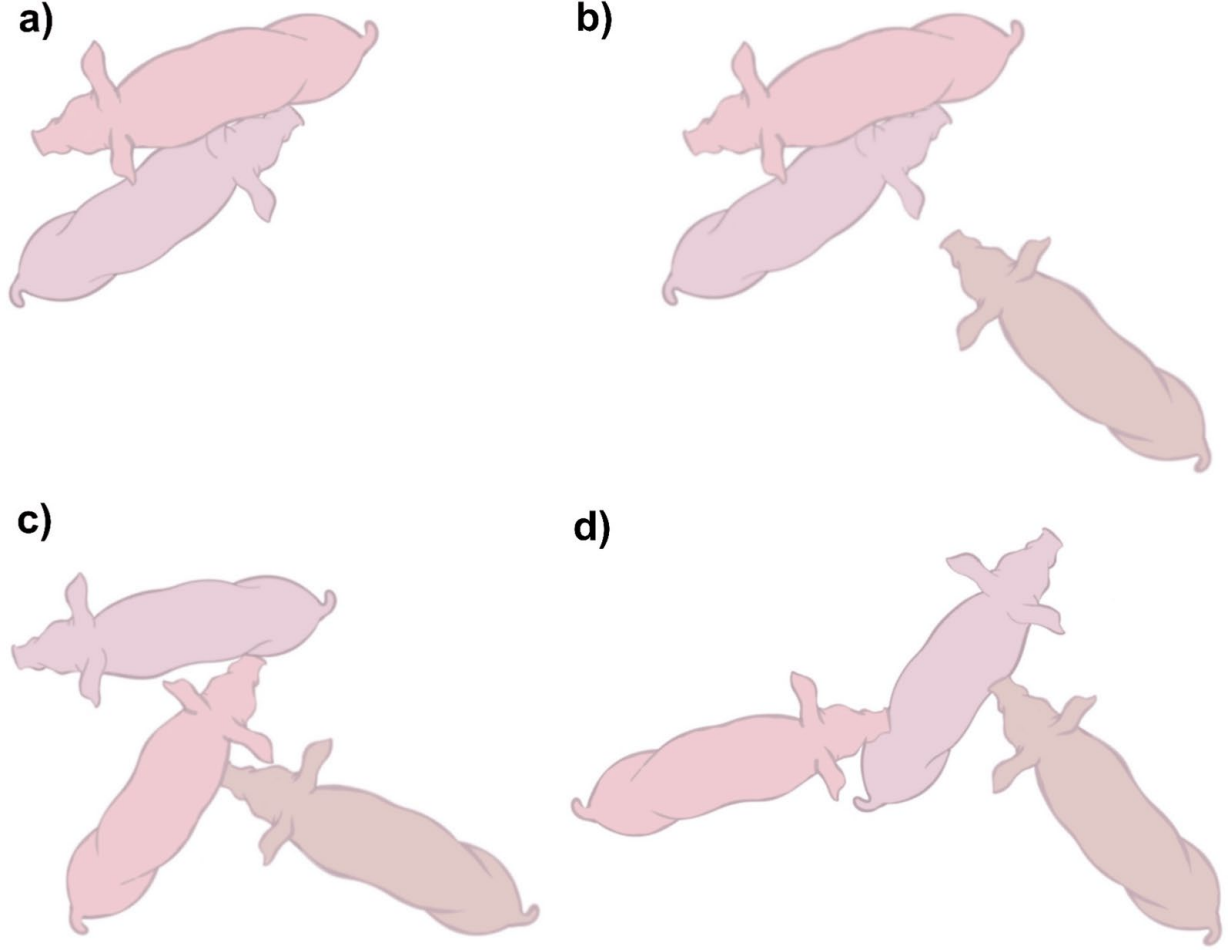
Types of interference. Fights were classified into **a **dyadic fights, **b** triadic interactions with a dyadic fight in which the interferer approached and nosed the opponent(s) but did not act aggressively, **c** triadic fights where the interferer (right pig) attacked one or both opponents, or **d** triadic fights in which two attacked one opponent simultaneously (i.e., two-against-one), with the interferer being attacker or attacked by the original opponents

**Fig. 2:**
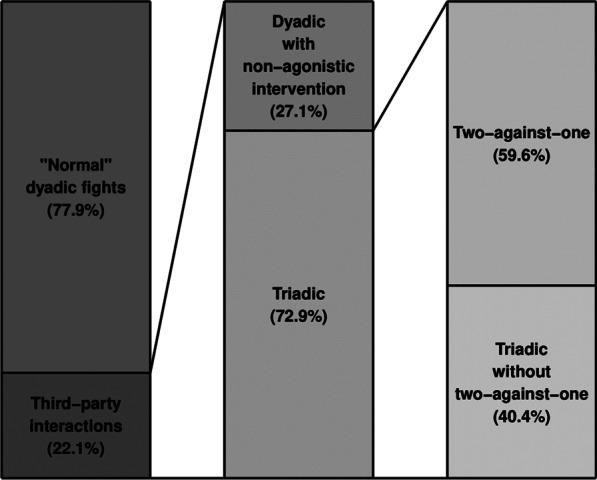
Types of aggressive interactions. Percentages relative to the type of interventions observed out of the total of 585 fights. In dyadic fights with non-agonistic intervention, the bystander only engaged with nosing, while in triadic fights it aggressively took part in the contest. Two-against-one fights refer to interactions when two of the opponents jointly attacked the one remaining opponent

During the first 30 min after the pairs of pigs were introduced to each other (six individuals per group), 585 fights were observed across the 64 different groups. Fights could be classified into dyadic fights, triadic interactions whereby the interferer approached and nosed the opponent(s) but did not engage with aggression, triadic fights where the interferer attacked one or both opponents (one at a time), or fights in which two pigs attacked one opponent simultaneously (i.e., two-against-one) (Fig. 1). Out of the total number of fights, 22.1% (n = 129/585) fights involved some form of third-party interference (Fig. 2). Nineteen fights (14.7%) involved two bystanders in succession, resulting in total in 129 different observations analysed. In most groups, only one or two fights involving a third party occurred (65% of the groups). The maximum number of episodes of third-party interference per group was seven (occurring in two groups). Bystanders more often intervened with aggressive behaviour (n = 94; 16.1% of the total number of fights) than with non-agonistic behaviour (n = 35; 6.0% of total number of fights; χ^2^ = 26.98, df = 1, *p* < 0.001). Out of the triadic interactions with aggression, 56 fights led to two-against-one fights (Fig. 2).

The likelihood of interfering was not affected by the interferer’s sex (72 cases with a male interferer vs. 57 cases with a female interferer; Chi-square test: χ ^2^ = 1.74, df = 1, *p* = 0.19). The average body weight of the interferer (on average 1.01 ± 1.96 kg (mean ± SD) heavier than the group average) did not differ from the average weight of the opponent which initiated the dyadic fight (i.e., fight initiator) (Welch two-sample t-test: t = 0.38, 95% CI [− 0.82, 1.21], df = 256, *p* = 0.71) or the initial victim in the dyadic fight (Welch two-sample t-test: t = 0.64, 95% CI [− 0.74, 1.45], df = 251, *p* = 0.52). Most bystanders interfered only once (n = 63), while 29 of them intervened multiple times.

### Prediction 1: the interferer gains direct benefits through partial intervention

Considering all triadic fights, the interferer won 58.4% of the contests. When excluding the eight contests whose outcome remained undecided, as neither opponent signaled their submission, the likelihood of winning was higher for the interferer compared to the likelihood of either one of the initial opponents (Chi-square test: χ^2^ = 5.90, df = 1, *p* = 0.015).

**Fig. 3:**
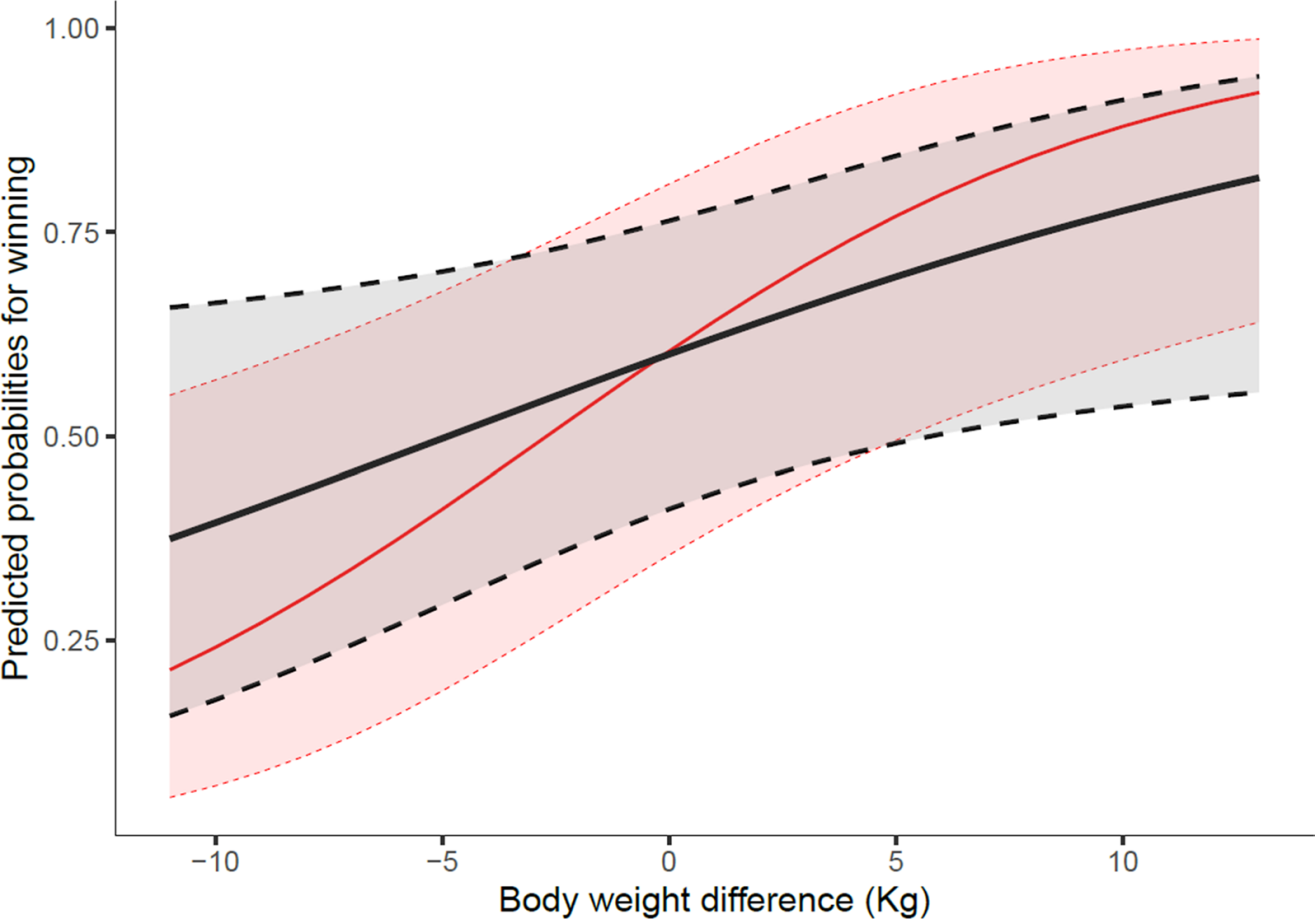
Probability of winning. Predicted probabilities (in black) of the interferer winning the fight plotted against body weight difference between interferer and its direct opponent. A positive body weight difference indicates that the interferer was heavier than its opponent. Dashed lines indicate the 95% confidence intervals relative to the probabilities. In red are the predicted probabilities of the fight initiator winning in a dyadic contest (without interference)

Interferers with a greater body weight than their opponent had greater odds of winning, independent of its sex (GLMM: β ± SE = 0.083 ± 0.040, 95% CI [0.01, 0.21], z = 2.07, *p* = 0.0380). When the interferer and its opponent were of the same body weight, the expected proportion of fights won by the interferer was 0.60 ± 0.39 (95% CI [0.41, 0.76]) (Fig. 3). However, even when the interferer was approximately 5 kg lighter than its opponent (with pigs being on average 32.6 ± 4.5 kg (mean ± SD)), the chance of winning was around 50% (Fig. 3). This indicates a clear benefit for the interferer’s chances of contest success.

### Prediction 2: interferers will predominantly aid the familiar individual

The interferer was familiar with one of the opponents in 46.5% of the cases (60 times), while unfamiliar in the rest of the cases (69 times). Due to the group composition, if interference was driven by chance, as the probability of the interferer being familiar with one of the two opponents in a dyadic fight would be 2/5. Given the observed probabilities, it seems that familiarity with one of the two initial opponents does not affect the odds of intervention (proportions test: χ^2^ = 2.28, df = 1, 95% CI [0.38, 0.55], *p* = 0.13).

**Fig. 4:**
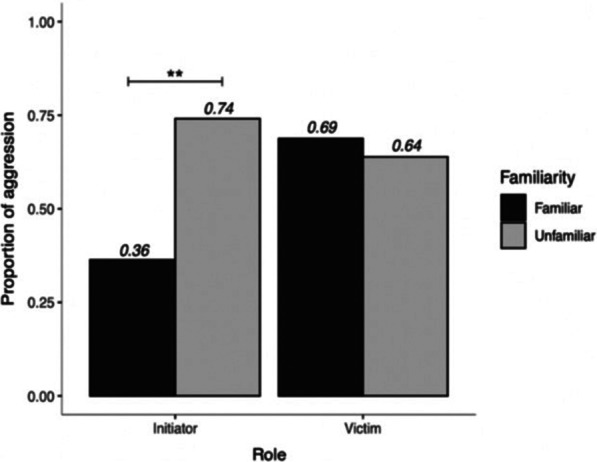
Role of familiarity. Proportion of aggressive acts shown by the bystanders towards the initiator and victim of the original dyadic fight, according to familiarity. Proportions are calculated as the number of times the interferer attacked the initiator/victim divided by the total number of third-party interactions. Numbers in italics represent the calculated proportions. Asterisks show the significance of the association between familiarity and behaviour of the interferer based on the two-proportions Z-test

Familiarity with one of the opponents did not influence the type of intervention in the fight, i.e., nosing vs. agonistic interaction as described in Fig. 1 (GLMM: β ± SE = [− 0.50 ± 0.46, 95% CI [− 1.53, 0.43], z = − 1.10, *p* = 0.27). Interferers interfered with only nosing (dyadic fights with or without familiar opponent: 31.7% vs. 23.3%, respectively) or with aggression (triadic fights with or without familiar opponent: 68.3% vs. 76.8%). However, interferers responded more often aggressively if the initiator of the original dyadic fight was unfamiliar (frequency of aggression towards an unfamiliar initiator: n = 60/81) than to a familiar initiator (n = 8/22) (Two-proportions Z-Test: χ^2^ = 9.35, 95% CI [− 0.63, − 0.13], df = 1, *p* = 0.002). Familiarity did not influence aggression towards the initial recipient (frequency of aggression towards a familiar recipient: n = 11/16, towards an unfamiliar recipient: n = 53/83) (Two-Proportions Z-Test: χ^2^ = 0.008, 95% CI [− 0.24, 0.34], df = 1, *p* = 0.93) (Fig. 4). Interferers behaved similarly to fight initiators and recipients, with initiators and recipients being equally nosed (in 13 and 15 cases, respectively) and attacked (in 72 and 68 cases, respectively) by the interferer. Sex and body weight of the interferer were not significant predictors of the occurrence of aggression (Sex: GLMM: β ± SE = 0.44 ± 0.52, 95% CI [− 0.55, 1.56], z = 0.84, *p* = 0.40; body weight: GLMM: β ± SE = 0.09 ± 0.06, 95% CI [− 0.03, 0.25], z = 1.47, *p* = 0.14).

Out of the 56 two-against-one fights, 41.1% (n = 23) of the triads included a pig familiar with the interferer. If random, the probability of a two-against-one fight involving a pig familiar to the interferer (2/5) would be 1.5 times lower than the chance of unfamiliarity (3/5). Therefore, the interferer’s likelihood of engagement in a two-against-one fight was not affected by familiarity (proportions test: χ^2^ = 0.03, df = 1, 95% CI [0.29, 0.54], *p* = 0.87). Considering only the two-against-one fights with a familiar individual, the interferer mostly attacked both opponents (52.2% of fights), or exclusively the unfamiliar opponent (43.5% of fights), while in only 4.3% (1 fight) exclusively attacked its familiar companion.

In accordance with the proportions test result, familiarity did not significantly affect the odds of a two-against-one fight (GLMM: β ± SE = 0.69 ± 0.53, 95% CI [− 0.44, 2.50], z = 1.31, *p* = 0.19). Sex and body weight relative to the opponent did not significantly relate to the occurrence of two-against-one fights. However, the likelihood of two-against-one fights was lower when the initial opponents (initiator and recipient) were of opposite sex, i.e., male–female, as compared to when they were both females (GLMM: β ± SE = -2.34 ± 0.66, 95% CI [− 15.07, − 1.28], z = -3.53, *p* < 0.001). No significant difference was found for the likelihood of two-against-one fights between male-male contests.

### Prediction 3: third party interference reduces the fight duration

**Fig. 5:**
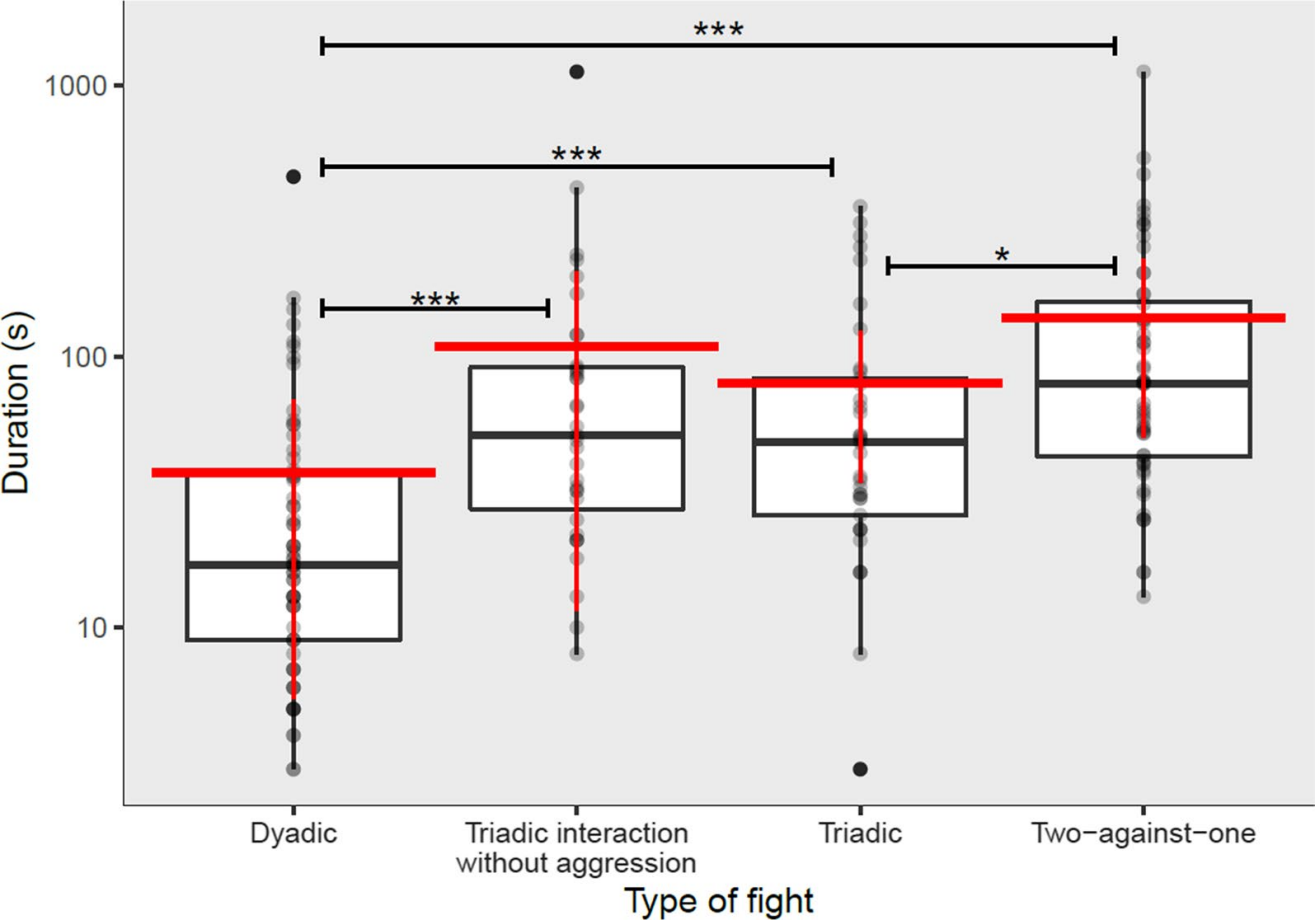
Fight duration. Boxplots of the duration (in seconds) for each type of fight. Boxes span the range from the first to the third quartile and the thicker line inside represents the median. Whiskers below and above the boxes show the minimum observation above the lower fence (Q1 − 1.5 IQR) and the maximum observation below the upper fence (Q3 + 1.5 IQR). Outliers are represented as solid circles. Data of individual fights are visible as dots. The red horizontal lines represent duration means and the red vertical lines represent standard deviations relative to the means. Asterisks show the significance of the differences based on the Linear Mixed Model and the Welch two-sample t-test

All types of triadic interactions resulted in a longer fight duration (mean ± SD: 111.8 ± 163.2 s) than dyadic fights without interference (mean ± SD = 37.1 ± 64.9 s) (Welch two sample t-test: *t* = [− 4.52, df = 183.1, *p* < 0.001, 95% CI [− 107.4, − 42.1]) (Fig. 5). Therefore, the prediction that interference may reduce contest duration and potentially aid group stability was not confirmed. Fight duration was however highly variable, with a minimum of 3 s and a maximum of over 18 min. Triadic fights without two-against-one were shorter than two-against-one fights (LMER contrasts: β ± SE = [− 0.59 ± 0.23, 95% CI [− 1.12, − 0.05], df = 113, t = − 2.064, *p* = 0.028) (Fig. 5). Familiarity did not predict the duration of the fight (LMER: β ± SE = 0.02 ± 0.19, 95% CI [− 0.35, 0.38], t = 0.10, *p* = 0.13).

### Prediction 4: interferers engage in post-conflict non-agonistic social behaviour

**Fig. 6:**
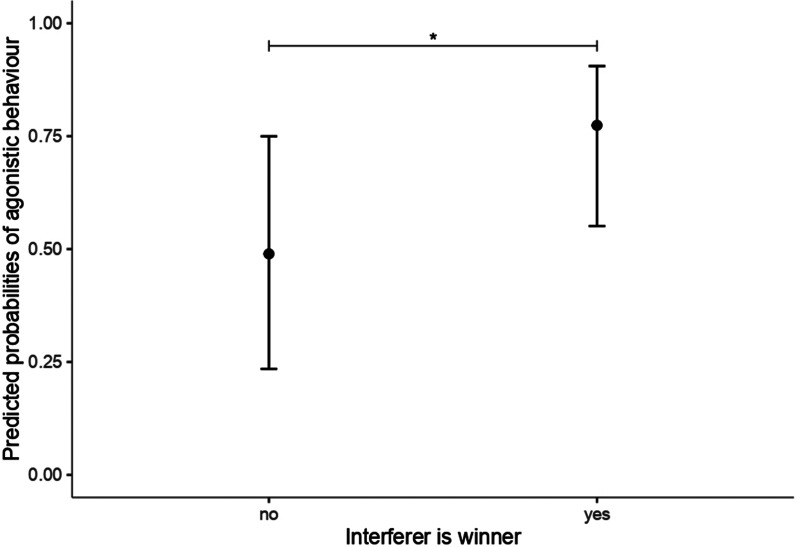
Post-conflict behaviour. Predicted probabilities of the interferer displaying post-conflict agonistic behaviour, based on whether it won or lost the preceding fight. Whiskers refer to 95% confidence intervals. The asterisk shows the significance level based on the Generalised Linear Mixed Model results

Following the conflict, the interferers which had engaged with aggression during the fight (as in the case of triadic fights and two-against-one fights) mostly behaved aggressively towards any other group mate (65.9% of the cases, 56 times), or performed non-social behaviour (29.4%, 25 times). Only in 4.7% of the cases (4 times), did the interferer engage in non-agonistic social interactions. The recipients of any social behaviour were mostly unfamiliar to the interferer (82.1%), indicating no preferential treatment towards the familiar companion (proportions test: χ^2^ = 0.21, df = 1, 95% CI [0.11, 0.28], *p* = 0.65). The outcome of triadic fights did affect the interferer’s post-conflict behaviour. When the interferer won, it was more likely to show social (agonistic or non-agonistic) than non-social behaviour, as compared to when it had lost (GLMM: β ± SE = 1.08 ± 0.54, 95% CI [0.05, 2.77], z = 2.02, *p* = 0.044). Accordingly, winning the fight increased the odds of the interferer showing aggression towards group mates directly after the conflict (GLMM: β ± SE = 1.27 ± 0.51, 95% CI [0.32, 2.99], z = 2.50, *p* = 0.012) (Fig. 6).

## Discussion

This study addressed third party interactions and fighting patterns of pigs upon being regrouped with unfamiliar individuals while remaining with one familiar conspecific. The aim of the study was to assess the potential benefits gained by a third-party from intervention. In this artificial regrouping scenario, which elicits pigs’ natural aggressive behaviour to establish dominance relationships, there was a high occurrence of triadic contests, including contests where two opponents fought simultaneously against one. The group setting, in which they had the choice regarding whether to fight or not, and who to fight with, shows that proactive interference in contests by pigs is not a rare occurrence. Our data showed that the interferer was more likely to win than both the initiator and recipient of the original fight and that intervention did not depend on the involvement of a familiar individual in the conflict. Future studies may be able to clarify whether interference is driven by the motivation of an individual to win an aggressive encounter or to specifically support one of the two contestants.

### Benefits of interference

Participation in a fight is costly in terms of both time and energy, and therefore interference is expected to be appropriately compensated by a net gain in terms of fitness (direct or indirect), and thereby the potential benefits should outweigh the costs [20, 95]. The proportion of third-party interactions in the current study relative to dyadic interactions is higher than that reported for other ungulates (e.g., *Dama dama*: 10% [42]) and even some primate species (e.g., *Papio cynocephalus*: 4–6% [85]). It should however be pointed out that domesticated animals were studied, in which fitness outcomes do not rely exclusively on natural selection, and artificial selection for production performance may have inadvertently increased aggressive behavior [23, 75]. Third party interactions occurred while two pigs were fighting in the close proximity of the third pig, and when interferers responded proactively by approaching the contestants.

Most interactions resulted in a win for the interferer, thus supporting the prediction that direct benefits would be gained through intervention. As expected, the rate of success increased if the interferer was heavier than its opponent, in accordance with the common usage of body weight as a proxy for the resource-holding potential (RHP) of an individual [2]. However, the fact that, even at equal weights, the interferer was more likely to win suggests that physical prowess is not an exhaustive explanation for the observed outcome, and interferers had a 50% chance of winning even when at a 5 kg weight disadvantage, which equates to a 15.3 ± 0.9% difference in mean body weight. It was previously shown that even a small weight disadvantage (0.5 kg), between pigs of the same age as in the current study, may result in losing [14].

When interference takes place, the initial opponents may already be in a state of fatigue, since they started fighting earlier than the interferer [61, 62]. Fatigue has an effect on the performance of the contestants and reduces the ability to fight [24]. The decreased stamina of the opponents may thus affect the outcome of the contest, giving an advantage to the interferer [9]. Higher motivation may also provide an explanation for how smaller opponents are able to succeed over larger individuals despite the physical disadvantage [39, 50]. Such willingness to fight may ultimately depend on the interaction of a variety of individual factors, which include aspects of personality and subjective resource value [10, 28, 44]. Furthermore, game-theoretical models show that, when there is no scarcity of resources, showing aggressiveness may yield more benefits to smaller individuals relative to larger animals for which it may be more convenient to avoid conflict and find alternative resources [61, 62]. In a range of species, social eavesdropping also appears to play a role whereby the bystander is able to infer the fighting abilities of the initial contestants and accordingly proceed with the intervention only when it judges that the odds are in its favour [2, 46, 90]. However, it is uncertain if this is applicable to our case, as it seems that pigs are not able to indirectly evaluate fighting abilities [65, 77], even at large weight differences [15].

Winning may result in a direct increase in dominance rank, subsequently affecting mating success and access to resources [1]. At the same time, the beneficial outcome of an intervention may result in a “winner effect”, that is the increased probability of winning a subsequent context, given a previous win [25, 26]. Winner-loser effects have been widely studied especially in controlled experimental conditions [34, 55], including in pigs [65]. An insight into the winner effect in the context of agonistic interference comes from Jennings et al. [42, 43], who investigated the relationship between third-party intervention and winner-loser effects in free-ranging fallow deer (*Dama dama*). Here the interference in a fight may serve the purpose of limiting the beneficial effects of winning for either of the original opponents, therefore preventing these animals from further increasing their status at its expense (“assurance of dominance hypothesis”) [92]. Accordingly, suffering a third-party intervention was found to reduce mating probability, at least in the short term [45]. Future studies may explore in more detail the potential long- term benefits derived from interference, for example taking into consideration changes in social status or in access to resources.

### Role of familiarity in interference

Familiarity with one of the opponents did not seem to influence the likelihood of a bystander’s participation in the fight. Similarly, the intensity of interference (e.g., whether the interference involved non-agonistic nosing or aggression) did not depend on familiarity with one of the opponents. This is in contrast with the expectations, as multiple previous studies have shown that provision of agonistic aid in a fight in favour of kin or known individuals is common in group-living species [17, 54, 87]. Moreover, even though the animals involved in the experiment were not genetically related, pigs appear to be able to form preferential associations [36]. Specifically, we previously showed that within the studied time frame these pigs showed a preference to remain in close proximity to the familiar conspecific and spaced away from the unfamiliar ones [13]. In addition to this, the possibility that familiar individuals may be perceived as kin and be treated accordingly cannot be completely ruled out, given that previous studies suggest recognition in pigs is based on familiarity and not genetic relatedness [72, 88].

Interferers were, however, more likely to attack unfamiliar fight initiators. This tendency of the interferer to be more willing to attack an unfamiliar initiator might be seen as a way to maximize the benefits of fighting without incurring unnecessary costs. Initiators are usually expected to be more likely to win as they pro-actively engage in the fight, while victims have to suffer the attack [41, 58, 70]. By preferentially targeting the unfamiliar initiator, the interferer may be able to test its strength with an unknown rival, and at the same time, by avoiding targeting familiar initiators, it may avoid wasting resources on known pigs, with whom a dominance relationship has already been established. It has been reported for other species that interferers preferentially aid the aggressor over the recipient, possibly to minimise the costs of fighting and to stabilise the group hierarchy [32]. The lack of relationship between familiarity with the recipient and likelihood of aggression, on the contrary, does not agree with what was found in other species (e.g., geladas: *Theropitecus gelada*), where bystanders preferentially support recipients, seemingly to reduce aggression within the group [68].

### Two-against-one fights

Two-against-one fights constituted the largest proportion of the total triadic contests. This implies that, at least temporarily, two of the three individuals involved in the fight simultaneously attacked the other one. In some species, joint attacks towards a common target may involve temporally stable “alliances”, reflective of long-term relationships between group members, or in other cases recruitment of temporary helpers based on certain useful characteristics, such as a high rank [40, 63, 84]. We hypothesized that the interferer would preferentially offer agonistic aid to familiar individuals but, from the lack of effect of familiarity on the occurrence of two-against-one fights as well as the lack of support to either the fight initiator or recipient, pigs do not seem to support a certain individual against the other. However, in more than 40% of the interactions the interferer attacked only the unfamiliar pig and only once did the interferer exclusively target the familiar individual. Even though, from our data, the lack of effect of familiarity on the occurrence of two-against-one fights seems to rule out the social support hypothesis, while familiarity seems to at least partially inhibit aggression.

There may be a range of reasons why an interferer targets one opponent rather than another. For example, spotted hyenas, gregarious carnivores with complex primate-like societies, seem to support the most dominant individual even when it is losing the fight, thus stabilizing the social rank of the group members (“group stability hypothesis”) [27]. Freeman et al. [33] proposed an alternative explanation based on mutualism: male vervet monkeys (*Chlorocebus pygerythrus*) appear to opportunistically form coalitions in order to take on a common opponent, all the while buffering the risks that fighting inherently entails. It is important to note that, even within the same species, third-party intervention may be driven by different mechanisms across contexts, as was proposed for Przewalski horses (*Equus ferus przewalskii*), where agonistic intervention promotes social cohesion in standard social situations but is also a means for formation of bonds when new group members are introduced [51]. The drivers of interference may ultimately depend on a unique set of costs and benefits, varying among individuals and situations, which make an animal more or less likely to intervene [1, 8, 44].

When a familiar pig was present in the triad, the fact that the interferer attacked both opponents on more than half of the occasions hints that the two initial contestants sometimes joined forces against the interferer. This is in accordance with the mutualistic hypothesis, since the interferer seems from the previous result to have an advantage over both opponents of the initial dyadic contest. Joint attacks would then be favoured by a large asymmetry in the fighting strength between opponents and interferer, with the unfavored opponents being more likely to attack jointly in order to increase their odds of winning [47, 63].

Female-preferential coalitions have been observed in primates, as in the case of wild female bonobos (*Pan paniscus*) or rhesus macaques (*Macaca mulatta*) [52, 91]. In this study, sex of the interferer was not a significant predictor of most of the studied variables. Although a confounding effect may come into play due to familiar dyads being always a male–female dyad, interferers were more likely to intervene when the initial fight initiator and victim were both females, as compared to a male–female dyad. Pigs show profound sex differences in their agonistic behaviour, with males showing more ritualized aggression and a superior fighting ability compared to females [16], and the lack of sex effects may in part be due to the fact that the males in the current study were castrated [78].

### Duration of the fight

While triadic interactions lasted longer than dyadic fights, there was no difference in duration between non-agonistic intervention in a dyadic fight and triadic fights. Intervention therefore does not seem to occur to bring fights to a swifter end and increase group stability.

Impartial intervention (or “policing”) occurs when a third-party intervenes in an on-going fight without partial treatment towards any participant, and it is thought to act as a means to manage conflict, thus promoting group cohesion [5, 92]. This type of intervention (shown in example Video S1, https://youtu.be/hyCD4jEjXsw; animals not part of this study) therefore constitutes a behaviour from which all group members (including the interferer) benefit [30]. According to the group stability hypothesis we would expect triadic fights to be shorter than dyadic fights, as the interferer would intervene in order to prevent the conflict from escalating and limit the damage caused by the fight [30]. However, policing is a rare behaviour, for now mostly observed in primates [92], and does not seem to be the function in the current study. The longer interaction duration may be partly due to additional dyadic interactions in a triadic involvement, as well as the opportunity for opponents to regain energy for a short period while the other two opponents are fighting. This may result in the three opponents being energetically better capable to sustain the fight for longer. As duration was not counted by individual it is not possible to exclude that involvement of a third party might reduce the time spent fighting for each individual. Future studies should take these aspects into consideration.

We found two-against-one fights to last longer than triadic fights without two-against-one. This is somewhat unexpected, as the combined strength of two individuals against a single pig should lead to a quicker resolution of the conflict. This result may be explained by the fact that two-against-one conflicts may subsequently break up into dyadic fights, thereby increasing the total length of the interaction. Development of fights in this way would also argue against interference having a policing role in pigs.

### Post-conflict behaviour

Triadic interactions may persist even after a fight, for example with bystanders approaching one of the opponents to offer post-conflict affiliation, which may constitute an effective way to mitigate the consequences of a conflict in social species [96]. This has recently also been observed in pigs, where it has been suggested to serve as reconciliation [22]. In our case, non-agonistic social behaviour by the interferer made up only a small part of the observations. Previous studies show that former opponents engage in post-conflict affiliation with individuals they share a close relationship with, e.g., kin or social partners [67, 71, 83] or with weakly related pigs [22]. The current study differs in that it observed interactions of the interferer with any other group member, rather than only the prior opponents. Further, the first half hour of regrouping is intense and fatigue may have reduced non-agonistic social interactions during this time. Affiliative behaviour between the study animals was shown over a longer time frame of 24 h, especially between the familiar conspecifics [13]. It may be that in the initial phase of intense aggression, pigs focus on the establishment of dominance relationships, whereas in later phases of the social group formation affiliative behaviour may become of more relevance.

The most common post-conflict behaviour of the interferer was further aggression towards any other group mate, and the likelihood of the interferer behaving aggressively increased if the interferer had won the previous fight. This may be further evidence of the previously discussed “winner effect”, with winning a previous fight increasing the chances of success for the interferer, which would then take advantage of this “positive streak” [42].

## Conclusions

Third-party intervention enhances the likelihood of winning for the interferer when new groups of pigs are formed, thus providing a direct benefit to the interferer. Interferers were more likely to attack unfamiliar fight initiators, but not exclusively, resulting in two opponents against one. However, we found no evidence in support of either the social support hypothesis or the group stability hypothesis. Intervention and coalition formation are still understudied phenomena. The high occurrence of third-party interferences in the current study, within the short time frame after opponents’ initial encounter, provides a useful model to further increase our knowledge of third-party interference and coalition formation.

## Methods

### Animals and housing

Male (n = 193) and female (n = 191) domestic pigs (commercial genetic crossbred based on Great Yorkshire and Dutch Landrace) of 9 weeks of age (average body weight: 32.6 ± 4.5 kg (mean ± SD)) were studied over four cohorts, at research farm ‘De Haar’, Wageningen, the Netherlands. In the context of another study [13], they were from 4 weeks of age housed in groups of six unrelated pigs (total 64 groups), with each group balanced for sex (1:1 females and castrated males). For research questions unrelated to this study, half of the groups were housed in a barren pen and the other half in an enriched pen, both at a space allowance of 1.0–1.2 m^2^ per animal. The barren pens had a 60% solid concrete and 40% slatted floor with minimal enrichment (a chain with ball). Enriched pens had a solid floor with a deep litter bedding of straw and wood shavings. Animals had access to ad libitum water from a drinker, and ad libitum pelleted feed from a single space feeder. Ambient temperature was automatically regulated to be at 20 °C and lights were on between 07:00 and 19:00 h.

### Regrouping test

To study agonistic behaviour in a group setting, pigs were regrouped with unfamiliar conspecifics for 24 h. Regrouping is a common management practice, and conducting this procedure under research settings allows testing of interventions that may result in improved welfare for the pig population at large. One male and one female from each group were jointly relocated into an unknown pen where they were, within a maximum of 15 min, grouped with two similar pairs (a male and female familiar to each other) originating from two different groups. This resulted in groups of six, consisting of three male–female pairs that were unfamiliar to each other, but known to their paired partner. No full-sibs were grouped together to avoid the possible effect of genetic relatedness beyond familiarity. Individuals were recognized by a spray mark (MS Marking spray, MS Schippers) on their back. Video cameras were mounted above the pens and recorded the full 24 h. After 24 h, pigs were returned to their initial group. Skin lesions from fighting were treated with a cutaneous chlortetracycline aerosol spray (CTC spray, Eurovet Animal Health BV, Netherlands). For each individual, the body weight at 9 weeks of age was known.

### Video observations

**Fig. 7:**
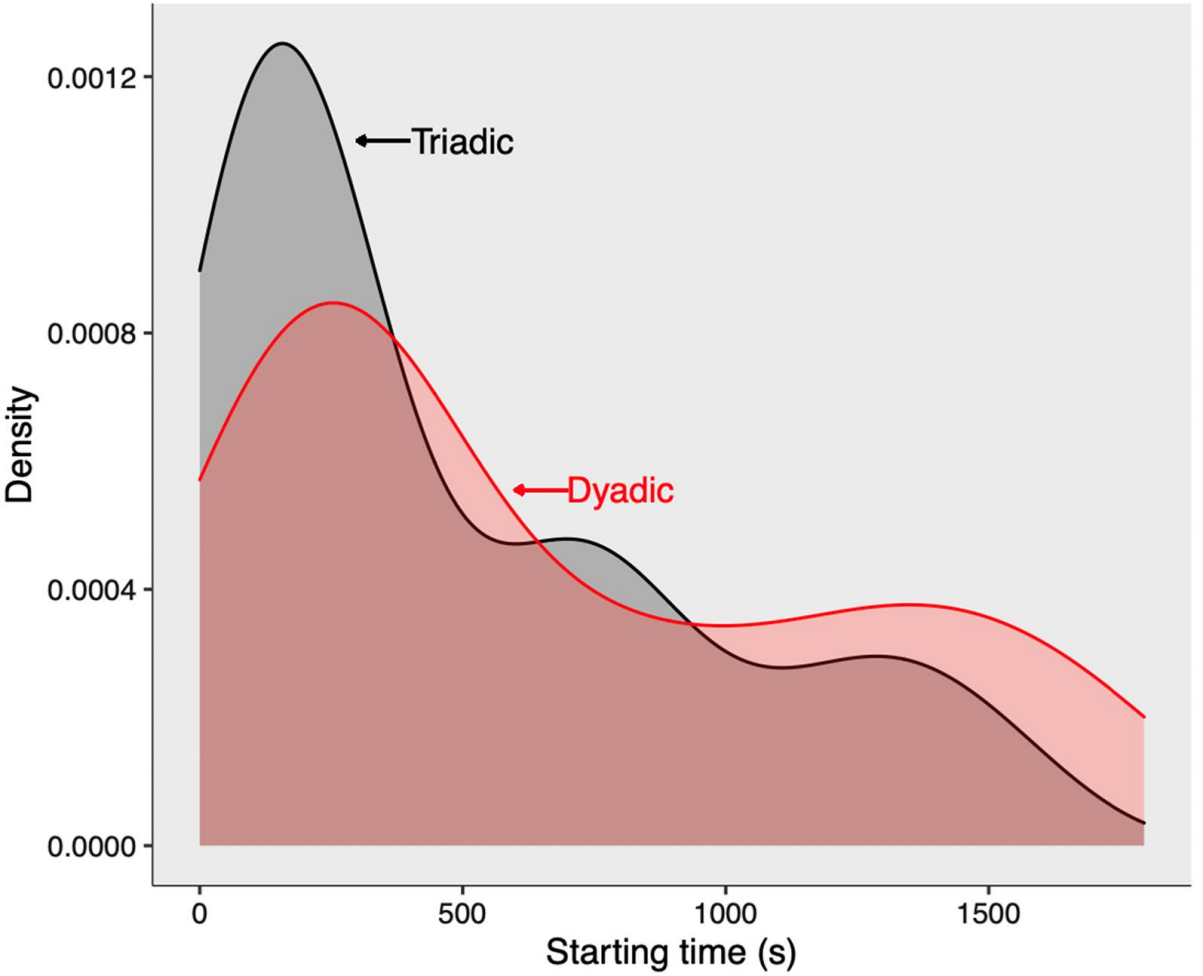
Distribution of dyadic and triadic fights. Density plot showing the distribution of dyadic fights (red) and triadic interactions (grey) during the 30 min after the beginning of the regrouping event (in seconds). Data is based on a subset of 12 randomly selected groups, totaling 66 fights, to determine the right time frame for observations

Behaviour was observed for the first 30 min after all pigs had entered the new pen. This timeslot captures the peak of the dyadic and triadic aggressive interactions, which typically occur when unfamiliar pigs meet. To assure us of the right time frame and duration, a subset of 12 randomly selected groups, totalling 66 fights, were observed for the timing and duration of dyadic and triadic fights (Fig. 7). The frequency of all dyadic and triadic fights was noted with the identity of the opponents. Fights were defined as a rapid sequence of bites which were retaliated against with an aggressive act from the opponent within 5 s. The winner of the fight was determined after one of the pigs retreated by showing a ‘head-tilt’ (a submissive behaviour) and subsequently did not retaliate. When the fight stopped for > 5 s and started again this was counted as a new fight. For fights where a bystander (third individual) participated, the fight duration in seconds was recorded from the start of the initial fight (between two opponents) until the resolution of the fight. Third party interference was recorded when the bystander proactively approached and made physical contact with one of the two fighting opponents (whether nose contact or aggression), along with the identity of the recipient. Based on the occurrence of behaviour, the interference of the bystander was labelled as ‘nosing’ or ‘aggression’. Social nosing, a behaviour that is mostly unrelated to agonistic behaviour in pigs [13], was defined as: the bystander approaches and touches, with its snout, the body or snout of one or both of the opponents. Aggression was defined as: the bystander bites or pushes one or both of the opponents, and either does or does not receive retaliatory aggression within 5 s.

After the initiation of a dyadic interaction, fights fell into one of the following four categories: (1) dyadic fights without interference; (2) dyadic fights with interference that involved only non-agonistic nosing by the bystander in the dyadic fight; (3) triadic fights where the bystander engaged in unilateral aggression or a fight in which each of the three opponents only fought one opponent at a given time (e.g., A attacks B, B attacks C and C attacks A); or (4) triadic fights in which two opponents jointly attacked another opponent causing a fight of two against one (e.g., A and B both attack C simultaneously), here referred to as ‘two-against-one’ (Fig. 1). In this case, the interferer may have been either one of the two pigs attacking together or may have ended up becoming the target of both original opponents. For 30 s after the fight terminated, the behaviour of the interferer was noted (Table 1) along with the identity of the recipient in the case of social behaviour. All observations were conducted by one observer, who showed 84.6% agreement with another observer when identifying third party interactions.

**Table 1:** Post-conflict behaviour of the interferer

Post-conflict behaviour of interferer	
Non-agonistic social behaviour	Interferer noses the body or snout of another pig, or lies in physical contact. The identity of the recipient is noted
Agonistic behaviour	Interferer delivers head knock, push, bite, or interferer fights with any group member, other than the former opponents. The identity of the recipient is noted
Non-social	Interferer stands, walks, lies individually, eats, drinks, urinates, defecates, or explores the environment

### Data analysis

Data were analysed in R (version 4.0.4) using two-tailed tests. The weight difference between interferer and initiator/recipient was studied with Welch two-sample t-tests.

As for the direct benefits hypothesis, the likelihood of the interferer winning (yes/no) was analysed only for the encounters including third-party interference, and excluding the fights with an undecided outcome (82 fights in total). This was analysed using Generalised Linear Mixed Models (GLMM: package *lme4*) [4] with a binary distribution and logit link. The predictor variables were (for each recorded social encounter) body weight difference between interferer and opponent, sex of the interferer, and familiarity of the interferer with at least one other opponent. To compute the predicted probability of winning from the GLMM, the package *ggeffects* was used [56]. A dataset containing the outcome of 65 dyadic fights (with no form of interference) was used to compare predicted probabilities of winning.

Occurrence of third-party interactions without aggression (nosing only) versus triadic fights was first studied with a chi-squared test. In accordance with the hypothesis of indirect benefits obtained through impartial intervention, to test whether the interfering individual targeted aggression towards familiar or unfamiliar opponents a two-proportions Z-test was used. Similarly, the probability of occurrence of triadic fights with or without two-against-one involving a pig familiar to the interferer was first explored through proportions tests. The likelihood of the occurrence of a fight involving aggressive interference (triadic or two-against-one) over a triadic interaction without aggression (nosing only) was then analysed for all encounters including third-party interference (124 encounters in total) using Generalised Linear Mixed Models (GLMM: package *lme4*; [4] with a binary distribution and logit link. The predictor variables were: sex of the interferer, body weight of the interferer, combined sexes of initiator and recipient (male-male (MM), female-female (FF), male–female (MF)), and presence of a pig familiar to the interferer in the triad. A subsequent model separately included familiarity to the initiator and to the recipient as predictors for the level of interference (nosing/aggression) (predictors: sex of the interferer, body weight of the interferer, combined sexes of initiator and recipient, presence of a familiar initiator, presence of a familiar recipient).

Similarly, the likelihood of occurrence of a two-against-one fight over a triadic fight was analysed only for encounters that included third-party interference (129 encounters in total), using Generalised Linear Mixed Models (GLMM: package *lme4*; [4] with a binary distribution and logit link. The predictor variables were (per triadic interaction): sex of the interferer, body weight difference between interferer and opponent, presence of a pig familiar to the interferer in the triad and combined sexes of initiator and recipient (male-male (MM), female-female (FF), male–female (MF)).

In order to test the group stability hypothesis (impartial intervention with indirect benefits), the duration of all types of triadic interactions was compared to the duration of dyadic interactions through a Welch two-sample t-test. Duration of the fights was analysed with Linear Mixed Models (LMER: package *lme4*; [4] with a lognormal distribution. The predictor variables of the model were: type of fight (triadic interaction without aggression/ triadic without two-against-one/triadic with two-against-one), combined sexes of initiator and recipient (MM, FF, MF), sex of the interferer, body weight difference between interferer and initiator/recipient, presence of a pig familiar to the interferer in the triad. A dataset comprising 65 dyadic fights was used to compare the duration of interactions with and without interference.

Post-conflict behaviour of the interferer (which had previously intervened with aggression) was analyzed with Generalised Linear Mixed Models (GLMM: package *lme4*; [4] with binary distribution and logit link. The dependent variables were: occurrence of agonistic vs. other behaviour (non-agonistic social and non-social) and occurrence of social (non-agonistic social and agonistic) versus non-social behaviour. The predictor variables were: sex and body weight of the interferer, type of conflict (triadic/two-against-one), and whether the interferer had won the fight (yes/no).

The observed probabilities of engagement with familiar individuals (for third-party interactions without two-against-one fight, third-party interactions with two-against-one fights, and post-conflict behaviour) were compared to the expected probabilities (calculated in accordance with the number of familiar and unfamiliar group mates) through proportion tests. Predictions of probabilities from the model were computed with the package *ggeffect* [56].

In all linear mixed models, group, nested within batch (cohort of 16 groups each), was included as a random factor to account for dependence between the group members, and dependence of the groups within a batch. Housing condition (barren/enriched) was included in each of the models but was omitted as it did not significantly relate to the response variables and reduced the model fit, as assessed through the AIC and BIC values. For multi-level categorical predictors, we ran *post-hoc* tests for pairwise comparisons (package *lsmeans*) [53].

The package *ggplot2* was used for data visualization [94]. Results are presented as the natural logarithm of the odds ratios with their confidence intervals (CI). Confidence intervals were calculated with bootstrapping (1000 simulations). Package *DHARMa* was used for model diagnostics [38]. The R script is made available in Additional file 1. 

### Supplementary Information

**Additional file1:** R script.

## Data Availability

The datasets used and/or analysed during the current study are available online under CC BY 4.0 license, https://doi.org/10.17632/3nw35p64my.1.
