# Supplementary material for: Third-party intervention and post-conflict behaviour in agonistic encounters of pigs (Sus scrofa)
Source: Front Zool. 2023 Aug 17;20:28. doi: 10.1186/s12983-023-00508-w (PMC10433626; doi:10.1186/s12983-023-00508-w)
Supplement: Supplementary file 1 — Additional file1: R script. [file 12983_2023_508_MOESM1_ESM.docx]

Third-party intervention and post-conflict behaviour in agonistic encounters of pigs (*Sus scrofa*)

Nicole Maffezzini^1, 2^, Simon P. Turner^2^, J. Elizabeth Bolhuis^3^, Gareth Arnott^4, *^, Irene Camerlink^1^

*^1^Institute of Genetics and Animal Biotechnology, Polish Academy of Sciences, Jastrzebiec, Poland; ^2^Animal Behaviour & Welfare, Animal and Veterinary Sciences Department, Scotland’s Rural College (SRUC), West Mains Rd., Edinburgh, EH9 3JG, UK; ^3^ Adaptation Physiology Group, Department of Animal Sciences, Wageningen University & Research, Wageningen, the Netherlands; ^4^ Institute for Global Food Security, School of Biological Sciences, Queen's University, Belfast, BT9 7BL, UK*

**R script (2022-11-06)**

Libraries

library(tidyverse)

library(lme4)

library(car)

library(ggeffects)

library(DHARMa)

library(lsmeans)

library(ggplot2)

library(parameters)

Triadic fights data

data_3_party <- readxl::read_xlsx("data-3rd-party-2021-1.xlsx")

data_3_party <- data_3_party %>% mutate(BW_diff_C = ifelse (C_wins_excl_undecided == 1, (BW_C – BW_loser), (BW_C – BW_winner)))

data_3_party <- data_3_party %>% mutate(sex_combined = as.factor(ifelse(SexA == SexB, paste(SexA, SexA, sep = ""), "FM")))

data_3_party <- data_3_party %>% mutate(Cfam_combined = ifelse(CfamA == CfamB, paste(CfamA, CfamA, sep = "-"), "FAM-UNFAM"))

data_3_party <- data_3_party %>% mutate(fam = ifelse(Cfam_combined == "FAM-UNFAM", "fam", "unfam"))

data_3_party <- data_3_party %>% mutate(CtoA = ifelse(Behav == "nosing", 0, CtoA)) %>% mutate(CtoA = ifelse(CtoA == 2, 1, CtoA))

data_3_party <- data_3_party %>% mutate(CtoB = ifelse(Behav =="nosing", 0, CtoB)) %>% mutate(CtoB = ifelse(CtoB ==2, 1, CtoB))

data_no_nosing <- data_3_party %>% filter(Behav == "Agg")

data_no_undecided_outcome <- data_3_party %>% filter(C_wins_excl_undecided != "NA")

Dyadic fights data

data_dyadic <- readxl::read_xlsx("data-extra-dyadic-fights-22.xlsx", skip = 1)

data_dyadic <- data_dyadic %>% mutate(dff_bw = BW_initiator – BW_opponent)

data_dyadic <- data_dyadic %>% mutate(initiator_wins = as.factor(ifelse(initiator == winner, "yes", "no")))

**1. The interferer gains direct benefits through partial intervention**

Model: win by the interferer

m_win <- glmer(C_wins_excl_undecided ~ BW_diff_C + SexC + fam +

(1|Batch/Batchpen),

data = data_no_nosing,

family = binomial(link = "logit")),

control = glmerControl(optimizer = 'optimx', optCtrl = list(method

= 'L-BFGS-B')))

confint(m_win, method = "boot", nsim = 1000)

pred_m_win <- ggpredict(m_win, terms = "BW_diff_C[-11:13]")

Outcome of dyadic fights

m_dyadic <- glmer(initiator_wins ~ dff_bw + (1|batch/pen),

data = data_dyadic,

family = binomial(link = "logit"))

pred_m_dyadic <- ggpredict(m_dyadic, terms = "dff_bw[-11:13]")

**2. Interferers will predominantly aid the familiar individual**

Model: behaviour of the interferer (without distinction between familiarity of initiator or opponent)

m_fam_nod <- glmer(as.factor(Behav) ~ SexC + sex_combined + BW_C + fam +

(1|Batch/Batchpen),

data = data_3_party,

family = binomial(link = "logit"))

lsmeans(m_fam_nod, pairwise ~ sex_combined)

confint(m_fam_nod, method = "boot", nsim = 1000)

Model: behaviour of the interferer (with distinction between familiarity of initiator or opponent)

m_fam_d <- glmer(as.factor(Behav) ~ SexC + sex_combined + BW_C + CfamA +

CfamB + (1|Batch/Batchpen),

data = data_3_party,

family = binomial(link = "logit"))

lsmeans(m_fam_d, pairwise ~ sex_combined)

confint(m_fam_d, method = "boot", nsim = 1000)

Model: occurrence of two-against-one fights

m_2vs1 <- glmer(as.factor(X2against1) ~ BW_diff_C + SexC + sex_combined +

Cfam_combined + (1|Batch/Batchpen),

data = data_no_nosing,

family = binomial(link = "logit"),

control = glmerControl(optimizer = 'optimx', optCtrl =

list(method = 'L-BFGS-B')))

lsmeans(m_2vs1, pairwise ~ sex_combined)

confint(m_2vs1, method = "boot", nsim = 1000)

**3. Third-party interference reduces the fight duration**

Preparing the data frame for the duration of the interactions

df_duration <- data[!is.na(data$duration), ]

df_duration <- df_duration %>% mutate(type = ifelse(X2against1 == 1, "Two-against-one", "Triadic"))

df_duration <- df_duration %>% mutate(type = ifelse(Behav == "nosing", "Dyadic", type))

df_duration <- df_duration %>% mutate(abs_BWC.A = abs(BW_diffC.A), abs_BWC.B = abs(BW_diffC.B))

Model: duration of the interaction

m_dur <- lmer(log(duration) ~ type + sex_combined + SexC + abs_BWC.A +

abs_BWC.B + Cfam_combined + (1|Batch/Batchpen),

data = df duration)

lsmeans(m_dur, pairwise ~ sex_combined)

confint(m_dur, method = "boot", nsim = 1000)

**4. Interferers engage in post-conflict non-agonistic social behaviour**

Preparing the data frames for post-conflict behaviour

df_pc_agonistic <- data_no_nosing %>% mutate(ago.noago = ifelse(post.behav.I == 3, 1, 0)) #agonistic behaviour = 1, non-agonistic behaviour = 0#

df_pc_social <- data_no_nosing %>% mutate(soc.nosoc = ifelse(post.behav.I == 1, 0, 1)) #social behaviour = 1, non-social behaviour = 0#

Model: agonistic vs non-agonistic post-conflict behaviour

m_ago <- glmer(ago.noago ~ SexC + BW_C + Incl_undecided + X2against1 +

(1|Batch/Batchpen),

data = df_pc_agonistic,

family = binomial(link = "logit"))

Model: social vs non-social post-conflict behaviour

m_soc <- glmer(soc.nosoc ~ SexC + BW_C + Incl_undecided + X2against1 +

(1|Batch/Batchpen),

data = df_pc_social,

family = binomial(link = "logit"))
